# Supplementary material for: Growth of complete ammonia oxidizers on guanidine
Source: Nature. 2024 Aug 14;633(8030):646–53. doi: 10.1038/s41586-024-07832-z (PMC11410670; doi:10.1038/s41586-024-07832-z)
Supplement: Supplementary file 1 — Supplementary Fig. 1 and Supplementary Tables 2–5 and 7–12. [file 41586_2024_7832_MOESM1_ESM.pdf]

nature portfolio

https://doi.org/10.1038/s41586-024-07832-z

Supplementary information

Growth of complete ammonia oxidizers on guanidine

In the format provided by the authors and unedited

Supplementary Information

Growth of complete ammonia oxidizers on guanidine

Authors: Marton Palatinszky[1], Craig W. Herbold[1,†], Christopher J. Sedlacek[1], Dominic Pühringer[2,3], Katharina Kitzinger[1], Andrew T. Giguere[1], Kenneth Wasmund[1,4††], Per H. Nielsen[4], Morten K. D. Dueholm[4], Nico Jehmlich[5], Richard Gruseck[1,6], Anton Legin[7], Julius Kostan[2,3], Nesrete Krasnici[2,3], Claudia Schreiner[2,3], Johanna Palmetzhofer[1,6], Thilo Hofmann[1], Michael Zumstein[1], Kristina Djinovíc-Carugo[2,3,8,9], Holger Daims[1,8], Michael Wagner[1,4,8*]

Affiliations

[1]Centre for Microbiology and Environmental Systems Science, University of Vienna, Vienna, Austria.

[2]Department of Structural and Computational Biology, Center for Molecular Biology, University of Vienna, Vienna, Austria.

[3]Max Perutz Labs, Vienna Biocenter Campus (VBC), Vienna, Austria.

[4]Center for Microbial Communities, Department of Chemistry and Bioscience, Aalborg University, Aalborg, Denmark.

[5]Helmholtz-Centre for Environmental Research-UFZ GmbH, Department of Molecular Systems Biology, 04318 Leipzig, Germany.

[6]Doctoral School in Microbiology and Environmental Science, University of Vienna, Vienna, Austria

[7]Institute of Inorganic Chemistry, Faculty of Chemistry, University of Vienna, Vienna, Austria

[8]The Comammox Research Platform. University of Vienna, Vienna, Austria.

[9]European Molecular Biology Laboratory (EMBL) Grenoble, France.

*Corresponding author. Email: michael.wagner@univie.ac.at

†Present address: Te Kura Pūtaiao Koiora - School of Biological Sciences, Te Whare Wānanga o Waitaha - University of Canterbury, Ōtautahi - Christchurch, Aotearoa - New Zealand

††Present address: School of Biological Sciences, University of Portsmouth, Portsmouth, UK.

Supplementary Figures

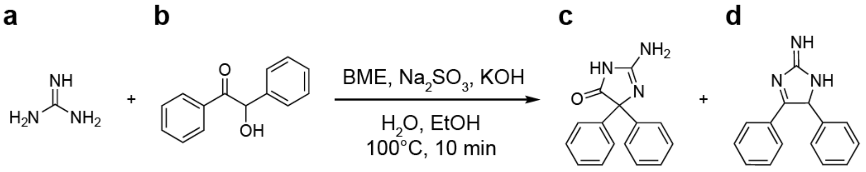

Supplementary Figure 1. Reaction scheme of the derivatization of guanidine (**a**) with benzoin (**b**). In addition to product **d**, which has been reported by Kai et al. based on fluorescence analysis[30], our analysis of the derivatization products by liquid chromatography coupled to mass spectrometry revealed another product (suggested structure **c,** (E)-1-(2-oxo-1,2-diphenylethylidene)guanidine), which we detected at a higher signal intensity than product **d**. In support of product **c**, which has previously been reported for a similar derivatization [96], we detected ions with the following exact mass-to-charge (m/z) ratios by high-resolution mass spectrometry: parent ion: 252.1131 (mass deviation: -1.74 ppm); fragment ions: 182.0964 (-1.79 ppm), 104.0495 (-1.69 ppm). Optimizing the derivatization protocol, we found that replacing 2-methoxyethanol with ethanol increased the fraction of product **c** (relative to product **d**) (Supplementary Table 5). BME: β-mercaptoethanol, EtOH: ethanol.

Supplementary Tables

Supplementary Table 1. Proteins proposed to be involved in guanidine degradation in ammonia oxidizing microorganisms. Shown are Genbank accession IDs for each genome examined, NCBI Bioproject, organism name according to NCBI, Type (comammox clade A, commamox clade B, gammaproteobacterial AOB-Nitrosococcaceae, betaproteobacterial AOB-Nitrosomonadaceae, or AOA) and classification according to genome taxonomy database (GTDB) version 207, according to which betaproteobacteria are classified as the order Burkholderiales within the gammaproteobacteria. For each protein, the scaffold accession ID, coordinates, orientation and protein accession ID are shown. In case more than one homolog was identified in a genome, the information within each cell is separated by a semicolon. **This table is provided as supplementary information as separate file in .xlsx (MS Excel) format.**

Supplementary Table 2. List of riboswitches detected in genomes of ammonia-oxidizing microorganisms. Each line represents a single riboswitch. If a genome assembly possessed more than one riboswitch, it is reported in separate lines.

Genbank assembly accession	Genbank Name	type	Bioproject	Scaffold accession	start	stop	orientation	type
GCA_001458695.1	*Nitrospira inopinata*	Comammox clade A	PRJEB10818	LN885086	1127535	1127638	+	ykkC-yxkD
GCA_001458735.1	*Candidatus* Nitrospira nitrosa	Comammox clade A	PRJEB11445	CZQA01000009	164697	164806	+	ykkC-yxkD
GCA_001458775.1	*Candidatus* Nitrospira nitrificans	Comammox clade A	PRJEB11446	CZPZ01000012	236418	236371	-	ykkC-III
GCA_001458775.1	*Candidatus* Nitrospira nitrificans	Comammox clade A	PRJEB11446	CZPZ01000023	114057	114161	+	ykkC-yxkD
GCA_014058405.1	*Candidatus* Nitrospira kreftii	Comammox clade A	PRJNA575653	CP047423	3752013	3751901	-	ykkC-yxkD
GCA_016722055.1	Nitrospira sp. (Ribe MAG)	Comammox clade A	PRJNA629478	JADKGV010000004	3470085	3469976	-	ykkC-yxkD
GCA_016712165.1	Nitrospira sp. (Haderslev MAG)	Comammox clade A	PRJNA629478	JADJRP010000001	193765	193874	+	ykkC-yxkD
GCA_001464735.1	Nitrospira sp. Ga0074138	Comammox clade A	PRJNA301005	LNDU01000015	54786	54837	+	ykkC-yxkD
GCA_001464735.1	Nitrospira sp. Ga0074138	Comammox clade A	PRJNA301005	LNDU01000042	102096	102206	+	ykkC-yxkD
GCA_002083365.1	Nitrospira sp. SG-bin1	Comammox clade A	PRJNA315256	LVWS01000031	96561	96670	+	ykkC-yxkD
GCA_002083365.1	Nitrospira sp. SG-bin1	Comammox clade A	PRJNA315256	LVWS01000043	119875	119807	-	ykkC-III
GCA_002083405.1	Nitrospira sp. SG-bin2	Comammox clade A	PRJNA315256	LVWT01000035	4476	4372	-	ykkC-yxkD
GCA_002083565.1	Nitrospira sp. ST-bin4	Comammox clade A	PRJNA315256	MSXM01000019	535	431	-	ykkC-yxkD
GCA_002254365.1	Nitrospira sp. UW-LDO-01	Comammox clade A	PRJNA322674	NIUT01000181	16249	16140	-	ykkC-yxkD
GCA_002331335.1	Nitrospira sp. UBA2082	Comammox clade A	PRJNA348753	DCZO01000014	280030	279981	-	mini-ykkC
GCA_002331335.1	Nitrospira sp. UBA2082	Comammox clade A	PRJNA348753	DCZO01000036	34961	35065	+	ykkC-yxkD
GCA_002420045.1	Nitrospira sp. UBA5702	Comammox clade A	PRJNA348753	DIHG01000065	42187	42078	-	ykkC-yxkD
GCA_003529185.1	Nitrospira sp.	Comammox clade A	PRJNA417962	DOJO01000045	61545	61436	-	ykkC-yxkD
GCA_005116745.1	Nitrospira sp.	Comammox clade A	PRJNA384587	SWDS01000002	328035	327982	-	mini-ykkC
GCA_005116745.1	Nitrospira sp.	Comammox clade A	PRJNA384587	SWDS01000003	8624	8513	-	ykkC-yxkD
GCA_005116895.1	Nitrospira sp.	Comammox clade A	PRJNA384587	SWDO01000005	407111	407227	+	ykkC-yxkD
GCA_005116895.1	Nitrospira sp.	Comammox clade A	PRJNA384587	SWDO01000014	167943	167996	+	mini-ykkC
GCA_005116955.1	Nitrospira sp.	Comammox clade A	PRJNA384587	SWDI01000005	813863	813973	+	ykkC-yxkD
GCA_005116965.1	Nitrospira sp.	Comammox clade A	PRJNA384587	SWDG01000005	13645	13533	-	ykkC-yxkD
GCA_009594825.1	Nitrospira sp.	Comammox clade A	PRJNA552823	VOPQ01000109	17497	17388	-	ykkC-yxkD
GCA_009595005.1	Nitrospira sp.	Comammox clade A	PRJNA552823	VOPO01000005	31726	31617	-	ykkC-yxkD
GCA_011090395.1	Nitrospira sp. LK265	Comammox clade A	PRJNA406858	PHGC01000255	515	619	+	ykkC-yxkD
GCA_011090425.1	Nitrospira sp. LK70	Comammox clade A	PRJNA406858	PHGD01000055	3262	3158	-	ykkC-yxkD
GCA_012032965.1	Nitrospira sp.	Comammox clade A	PRJNA612530	JAAUPT010000164	4174	4285	+	ykkC-yxkD
GCA_013141205.1	Nitrospira sp.	Comammox clade A	PRJNA622654	JABFRI010000329	4442	4332	-	ykkC-yxkD
GCA_015903945.1	Nitrospira sp.	Comammox clade A	PRJNA559097	JABMDL010000079	4912	4808	-	ykkC-yxkD
GCA_015903955.1	Nitrospira sp.	Comammox clade A	PRJNA559097	JABMDM010000033	16270	16374	+	ykkC-yxkD
GCA_015903995.1	Nitrospira sp.	Comammox clade A	PRJNA559097	JABMDK010000011	124953	125065	+	ykkC-yxkD
GCA_015904045.1	Nitrospira sp.	Comammox clade A	PRJNA559097	JABMDG010000028	14922	15026	+	ykkC-yxkD
GCA_015904045.1	Nitrospira sp.	Comammox clade A	PRJNA559097	JABMDG010000028	19069	19173	+	ykkC-yxkD
GCA_016715825.1	Nitrospira sp.	Comammox clade A	PRJNA629478	JADJXO010000003	75714	75823	+	ykkC-yxkD
GCA_016788465.1	Nitrospira sp.	Comammox clade A	PRJNA592128	JAEURV010000038	29445	29369	-	ykkC-yxkD
GCA_016789065.1	Nitrospira sp.	Comammox clade A	PRJNA592128	JAEURW010000152	43908	43797	-	ykkC-yxkD
GCA_016873455.1	Nitrospira sp.	Comammox clade A	PRJNA523022	VGXE01000032	3167	3063	-	ykkC-yxkD
GCA_018242685.1	Nitrospira sp.	Comammox clade A	PRJNA694732	JAFEBM010000013	14393	14289	-	ykkC-yxkD
GCA_018242765.1	Nitrospira sp.	Comammox clade A	PRJNA694732	JAFEBH010000025	29097	29208	+	ykkC-yxkD
GCA_018242825.1	Nitrospira sp.	Comammox clade A	PRJNA694732	JAFEBE010000009	179495	179384	-	ykkC-yxkD
GCA_019636815.1	Nitrospira sp.	Comammox clade A	PRJNA725625	JAHBWT010000038	2382	2272	-	ykkC-yxkD
GCA_019636835.1	Nitrospira sp.	Comammox clade A	PRJNA725625	JAHBWU010000003	93367	93471	+	ykkC-yxkD
GCA_019636915.1	Nitrospira sp.	Comammox clade A	PRJNA725625	JAHBWQ010000140	1086	975	-	ykkC-yxkD
GCA_019636945.1	Nitrospira sp.	Comammox clade A	PRJNA725625	JAHBWO010000003	35754	35863	+	ykkC-yxkD
GCA_019750685.1	Nitrospiraceae bacterium	Comammox clade A	PRJNA745370	JAIEOE010000028	7108	7212	+	ykkC-yxkD
GCA_022842445.1	Nitrospira sp.	Comammox clade A	PRJNA764197	JALHPO010000014	27066	27170	+	ykkC-yxkD
GCA_022843785.1	Nitrospira sp.	Comammox clade A	PRJNA764197	JALHMZ010000038	10440	10336	-	ykkC-yxkD
GCA_900696515.1	uncultured Nitrosospira sp.	Comammox clade A	PRJEB31362	CAADGK010000012	694	803	+	ykkC-yxkD
GCA_902500705.1	uncultured Nitrospira sp.	Comammox clade A	PRJEB30654	CABVRR010000033	11025	11081	+	mini-ykkC

GCA_902500705.1	uncultured Nitrospira sp.	Comammox clade A	PRJEB30654	CABVRR010000120	6087	6192	+	ykkC-yxkD
GCA_902500745.1	uncultured Nitrospira sp.	Comammox clade A	PRJEB30654	CABVRS010000180	331	440	+	ykkC-yxkD
GCA_902500755.1	uncultured Nitrospira sp.	Comammox clade A	PRJEB30654	CABVRV010000099	556	452	-	ykkC-yxkD
GCA_902500755.1	uncultured Nitrospira sp.	Comammox clade A	PRJEB30654	CABVRV010000099	5188	5084	-	ykkC-yxkD
GCA_902500755.1	uncultured Nitrospira sp.	Comammox clade A	PRJEB30654	CABVRV010000099	11105	11001	-	ykkC-yxkD
GCA_902500755.1	uncultured Nitrospira sp.	Comammox clade A	PRJEB30654	CABVRV010000108	562	451	-	ykkC-yxkD
GCA_902500775.1	uncultured Nitrospira sp.	Comammox clade A	PRJEB30654	CABVRZ010000063	9256	9135	-	ykkC-yxkD
GCA_902500795.1	uncultured Nitrospira sp.	Comammox clade A	PRJEB30654	CABVSB010000058	6582	6687	+	ykkC-yxkD
GCA_902500795.1	uncultured Nitrospira sp.	Comammox clade A	PRJEB30654	CABVSB010000058	15804	15909	+	ykkC-yxkD
GCA_902500805.1	uncultured Nitrospira sp.	Comammox clade A	PRJEB30654	CABVSA010000054	19235	19126	-	ykkC-yxkD
GCA_902500825.1	uncultured Nitrospira sp.	Comammox clade A	PRJEB30654	CABVRX010000004	85372	85267	-	ykkC-yxkD
GCA_902501035.1	uncultured Nitrospira sp.	Comammox clade A	PRJEB30654	CABVST010000376	2757	2648	-	ykkC-yxkD
GCA_919902665.1	Nitrospiraceae bacterium	Comammox clade A	PRJEB36523	CAKKRK010000101	194	304	+	ykkC-yxkD
GCA_005239475.1	Nitrospira sp.	Comammox clade B	PRJNA513947	SPAX01000080	7268	7377	+	ykkC-yxkD
GCA_005239475.1	Nitrospira sp.	Comammox clade B	PRJNA513947	SPAX01000256	16140	16035	-	ykkC-yxkD
GCA_013140715.1	Nitrospiraceae bacterium	Comammox clade B	PRJNA622654	JABFSG010000071	28334	28230	-	ykkC-yxkD
GCA_016219645.1	Nitrospirae bacterium	Comammox clade B	PRJNA640378	JACRLR010000021	21389	21431	+	mini-ykkC
GCA_919901595.1	Nitrospiraceae bacterium	Comammox clade B	PRJEB36523	CAKKPD010000002	101348	101452	+	ykkC-yxkD
GCA_919902055.1	Nitrospira bact. HGW-Nitrospira-1	Comammox clade B	PRJEB36523	CAKKQR010000533	1129	1025	-	ykkC-yxkD
GCA_000009145.1	Nitrosomonas europaea ATCC 19718	AOB - Nitrosomonadaceae	PRJNA52	AL954747	2389357	2389307	-	mini-ykkC
GCA_000009145.1	Nitrosomonas europaea ATCC 19718	AOB - Nitrosomonadaceae	PRJNA52	AL954747	2634203	2634101	-	ykkC-yxkD
GCA_000009145.1	Nitrosomonas europaea ATCC 19718	AOB - Nitrosomonadaceae	PRJNA52	AL954747	2634421	2634524	+	ykkC-yxkD
GCA_000014765.1	Nitrosomonas eutropha C91	AOB - Nitrosomonadaceae	PRJNA13913	CP000450	2598249	2598146	-	ykkC-yxkD
GCA_000014765.1	Nitrosomonas eutropha C91	AOB - Nitrosomonadaceae	PRJNA13913	CP000450	2598462	2598562	+	ykkC-yxkD
GCA_000196355.1	Nitrosospira multiformis ATCC 25196	AOB - Nitrosomonadaceae	PRJNA13912	CP000103	1087459	1087562	+	ykkC-yxkD
GCA_000355765.3	Nitrosospira lacus	AOB - Nitrosomonadaceae	PRJEB1373	CP021106	1542253	1542358	+	ykkC-yxkD
GCA_000619905.1	Nitrosospira briensis C-128	AOB - Nitrosomonadaceae	PRJNA183056	CP012371	796231	796128	-	ykkC-yxkD
GCA_000832065.1	Nitrosospira sp. NpAV	AOB - Nitrosomonadaceae	PRJNA272527	JXQM01000011	3465	3576	+	ykkC-yxkD
GCA_001007935.1	Nitrosomonas communis	AOB - Nitrosomonadaceae	PRJNA282404	CP011451	2425204	2425098	-	ykkC-yxkD
GCA_001455205.1	Nitrosomonas ureae	AOB - Nitrosomonadaceae	PRJNA304608	CP013341	1136991	1136887	-	ykkC-yxkD
GCA_001455205.1	Nitrosomonas ureae	AOB - Nitrosomonadaceae	PRJNA304608	CP013341	1443043	1443090	+	mini-ykkC
GCA_001567435.1	Nitrosomonas europaea	AOB - Nitrosomonadaceae	PRJNA274364	JZQZ01000027	1428	1478	+	mini-ykkC
GCA_001567435.1	Nitrosomonas europaea	AOB - Nitrosomonadaceae	PRJNA274364	JZQZ01000061	1	84	+	ykkC-yxkD
GCA_001567435.1	Nitrosomonas europaea	AOB - Nitrosomonadaceae	PRJNA274364	JZQZ01000114	121	223	+	ykkC-yxkD
GCA_001899235.1	Nitrosospira sp. 56-18	AOB - Nitrosomonadaceae	PRJNA279279	MKVR01000007	493	388	-	ykkC-yxkD
GCA_001899235.1	Nitrosospira sp. 56-18	AOB - Nitrosomonadaceae	PRJNA279279	MKVR01000049	11326	11431	+	ykkC-yxkD
GCA_002083395.1	Proteobacteria bacterium SG_bin4	AOB - Nitrosomonadaceae	PRJNA315256	LWDH01000045	77685	77787	+	ykkC-yxkD
GCA_002083595.1	Proteobacteria bacterium ST_bin16	AOB - Nitrosomonadaceae	PRJNA315256	MSXT01000012	30537	30433	-	ykkC-yxkD
GCA_003046585.1	Nitrosomonas aestuarii	AOB - Nitrosomonadaceae	PRJNA444064	QAAE01000011	23757	23894	+	ykkC-yxkD
GCA_003046585.1	Nitrosomonas aestuarii	AOB - Nitrosomonadaceae	PRJNA444064	QAAE01000016	11770	11726	-	mini-ykkC
GCA_003050865.1	Nitrosospira multiformis	AOB - Nitrosomonadaceae	PRJNA444074	QAOK01000008	54045	53941	-	ykkC-yxkD
GCA_003051045.1	Nitrosomonas ureae	AOB - Nitrosomonadaceae	PRJNA444081	QAOL01000002	47424	47528	+	ykkC-yxkD
GCA_003051045.1	Nitrosomonas ureae	AOB - Nitrosomonadaceae	PRJNA444081	QAOL01000014	58519	58566	+	mini-ykkC
GCA_008015595.1	Nitrosomonas sp.	AOB - Nitrosomonadaceae	PRJNA428383	SSFW01000135	10705	10594	-	ykkC-yxkD
GCA_008015635.1	Nitrosomonas oligotropha	AOB - Nitrosomonadaceae	PRJNA428383	SSFX01000064	1824	1719	-	ykkC-yxkD
GCA_008124795.1	Nitrosomonas communis	AOB - Nitrosomonadaceae	PRJNA330478	VNHT01000011	12347	12453	+	ykkC-yxkD
GCA_009833085.1	Nitrosomonas oligotropha	AOB - Nitrosomonadaceae	PRJNA284623	QRBZ01000012	61652	61547	-	ykkC-yxkD
GCA_009833095.1	Nitrosomonas sp. JL21	AOB - Nitrosomonadaceae	PRJNA284623	QRCB01000027	58314	58210	-	ykkC-yxkD
GCA_009833115.1	Nitrosomonas sp. GH22	AOB - Nitrosomonadaceae	PRJNA284623	QRCA01000038	15437	15334	-	ykkC-yxkD
GCA_009833115.1	Nitrosomonas sp. GH22	AOB - Nitrosomonadaceae	PRJNA284623	QRCA01000038	15650	15750	+	ykkC-yxkD
GCA_009833125.1	Nitrosomonas sp. HPC101	AOB - Nitrosomonadaceae	PRJNA284623	QRCC01000009	143352	143248	-	ykkC-yxkD
GCA_009833125.1	Nitrosomonas sp. HPC101	AOB - Nitrosomonadaceae	PRJNA284623	QRCC01000021	6689	6586	-	ykkC-yxkD
GCA_013450215.1	Nitrosospira sp.	AOB - Nitrosomonadaceae	PRJNA636190	JACDSH010000031	16059	15953	-	ykkC-yxkD
GCA_013697655.1	Nitrosospira sp.	AOB - Nitrosomonadaceae	PRJNA630822	JACCWK010000163	3563	3458	-	ykkC-yxkD
GCA_014584635.1	Nitrosomonas eutropha	AOB - Nitrosomonadaceae	PRJNA335309	JACTML010000032	10189	10090	-	ykkC-yxkD
GCA_014584635.1	Nitrosomonas eutropha	AOB - Nitrosomonadaceae	PRJNA335309	JACTML010000032	10630	10734	+	ykkC-yxkD
GCA_015075225.1	Burkholderiales bacterium	AOB - Nitrosomonadaceae	PRJNA627084	JABTUQ010000005	108848	108747	-	ykkC-yxkD
GCA_015075225.1	Burkholderiales bacterium	AOB - Nitrosomonadaceae	PRJNA627084	JABTUQ010000005	109076	109177	+	ykkC-yxkD
GCA_015709435.1	Nitrosomonas sp. H1_AOB3	AOB - Nitrosomonadaceae	PRJNA627084	CP054389	19260	19310	+	mini-ykkC
GCA_015709435.1	Nitrosomonas sp. H1_AOB3	AOB - Nitrosomonadaceae	PRJNA627084	CP054389	2498788	2498686	-	ykkC-yxkD
GCA_015709435.1	Nitrosomonas sp. H1_AOB3	AOB - Nitrosomonadaceae	PRJNA627084	CP054389	2499006	2499109	+	ykkC-yxkD
GCA_015709615.1	Gammaproteobacteria bacterium	AOB - Nitrosomonadaceae	PRJNA627084	CP054179	1081753	1081858	+	ykkC-yxkD
GCA_015709635.1	Gammaproteobacteria bacterium	AOB - Nitrosomonadaceae	PRJNA627084	CP054180	821049	820946	-	ykkC-yxkD
GCA_016106405.1	Nitrosospira sp.	AOB - Nitrosomonadaceae	PRJNA636190	JADMAR010000070	25168	25274	+	ykkC-yxkD
GCA_016106425.1	Nitrosospira sp.	AOB - Nitrosomonadaceae	PRJNA636190	JADMAG010000092	8349	8455	+	ykkC-yxkD
GCA_016106425.1	Nitrosospira sp.	AOB - Nitrosomonadaceae	PRJNA636190	JADMAG010000213	6965	6859	-	ykkC-yxkD
GCA_016703745.1	Nitrosomonas sp.	AOB - Nitrosomonadaceae	PRJNA629478	JADJBK010000006	628264	628147	-	ykkC-yxkD
GCA_016705625.1	Nitrosomonas sp.	AOB - Nitrosomonadaceae	PRJNA629478	JADJLE010000001	1830522	1830633	+	ykkC-yxkD
GCA_016707165.1	Nitrosomonas sp.	AOB - Nitrosomonadaceae	PRJNA629478	JADJIF010000025	109552	109657	+	ykkC-yxkD
GCA_016708955.1	Nitrosomonas sp.	AOB - Nitrosomonadaceae	PRJNA629478	JADJES010000001	745951	745846	-	ykkC-yxkD
GCA_016710135.1	Nitrosomonas sp.	AOB - Nitrosomonadaceae	PRJNA629478	JADJLM010000001	440252	440141	-	ykkC-yxkD
GCA_016717525.1	Nitrosomonas sp.	AOB - Nitrosomonadaceae	PRJNA629478	JADKFI010000019	39987	39883	-	ykkC-yxkD
GCA_016721865.1	Nitrosomonas sp.	AOB - Nitrosomonadaceae	PRJNA629478	JADKHF010000042	84437	84332	-	ykkC-yxkD
GCA_017306155.1	Nitrosospira multiformis	AOB - Nitrosomonadaceae	PRJNA629336	JAFKJB010000009	90947	91051	+	ykkC-yxkD
GCA_018240285.1	Proteobacteria bacterium	AOB - Nitrosomonadaceae	PRJNA694732	JAFEGA010000008	42724	42619	-	ykkC-yxkD
GCA_018240885.1	Proteobacteria bacterium	AOB - Nitrosomonadaceae	PRJNA694732	JAFEEW010000074	29830	29935	+	ykkC-yxkD
GCA_018241205.1	Proteobacteria bacterium	AOB - Nitrosomonadaceae	PRJNA694732	JAFEEI010000096	5503	5398	-	ykkC-yxkD
GCA_018406445.1	Nitrosospira sp. NRS527	AOB - Nitrosomonadaceae	PRJDB11303	AP024515	1047137	1047240	+	ykkC-yxkD
GCA_019186865.1	Nitrosomonas europaea	AOB - Nitrosomonadaceae	PRJNA559529	JABARC010000035	11606	11504	-	ykkC-yxkD
GCA_019186865.1	Nitrosomonas europaea	AOB - Nitrosomonadaceae	PRJNA559529	JABARC010000035	11824	11927	+	ykkC-yxkD
GCA_019186865.1	Nitrosomonas europaea	AOB - Nitrosomonadaceae	PRJNA559529	JABARC010000039	2617	2567	-	mini-ykkC
GCA_019187415.1	Nitrosomonas sp.	AOB - Nitrosomonadaceae	PRJNA559529	JABAQW010000036	3462	3567	+	ykkC-yxkD
GCA_019635155.1	Nitrosomonas sp.	AOB - Nitrosomonadaceae	PRJNA725625	JAHBZY010000017	31381	31280	-	ykkC-yxkD
GCA_019635175.1	Nitrosomonas sp.	AOB - Nitrosomonadaceae	PRJNA725625	JAHBZX010000001	41285	41391	+	ykkC-yxkD

GCA_019635175.1	Nitrosomonas sp.	AOB - Nitrosomonadaceae	PRJNA725625	JAHBZX010000035	20082	20038	-	mini-ykkC
GCA_019635215.1	Nitrosomonas sp.	AOB - Nitrosomonadaceae	PRJNA725625	JAHBZZ010000003	53988	54094	+	ykkC-yxkD
GCA_019635215.1	Nitrosomonas sp.	AOB - Nitrosomonadaceae	PRJNA725625	JAHBZZ010000006	116056	116012	-	mini-ykkC
GCA_019635215.1	Nitrosomonas sp.	AOB - Nitrosomonadaceae	PRJNA725625	JAHBZZ010000022	1147	981	-	ykkC-yxkD
GCA_019745325.1	Nitrosomonas sp.	AOB - Nitrosomonadaceae	PRJNA745370	JAIETK010000004	25042	24939	-	ykkC-yxkD
GCA_019745535.1	Nitrosomonas sp.	AOB - Nitrosomonadaceae	PRJNA745370	JAIETB010000046	6166	6062	-	ykkC-yxkD
GCA_020696875.1	Nitrosospira multiformis	AOB - Nitrosomonadaceae	PRJNA725542	JAJFMQ010000002	66565	66669	+	ykkC-yxkD
GCA_020852475.1	Nitrosomonas sp.	AOB - Nitrosomonadaceae	PRJNA611787	JADZAR010000060	768	721	-	mini-ykkC
GCA_020852475.1	Nitrosomonas sp.	AOB - Nitrosomonadaceae	PRJNA611787	JADZAR010000095	1	50	+	ykkC-yxkD
GCA_020890945.1	Nitrosomonas sp.	AOB - Nitrosomonadaceae	PRJNA691751	JAJHPK010000100	7477	7353	-	ykkC-yxkD
GCA_021462965.1	Nitrosomonas sp.	AOB - Nitrosomonadaceae	PRJNA791618	JAJTXG010000001	772093	771989	-	ykkC-yxkD
GCA_021462965.1	Nitrosomonas sp.	AOB - Nitrosomonadaceae	PRJNA791618	JAJTXG010000001	772534	772633	+	ykkC-yxkD
GCA_021463265.1	Nitrosomonas sp.	AOB - Nitrosomonadaceae	PRJNA791618	JAJTXH010000001	114369	114319	-	mini-ykkC
GCA_021463265.1	Nitrosomonas sp.	AOB - Nitrosomonadaceae	PRJNA791618	JAJTXH010000002	1773441	1773338	-	ykkC-yxkD
GCA_021463265.1	Nitrosomonas sp.	AOB - Nitrosomonadaceae	PRJNA791618	JAJTXH010000002	1773659	1773761	+	ykkC-yxkD
GCA_021463765.1	Nitrosomonas sp.	AOB - Nitrosomonadaceae	PRJNA791618	JAJTXI010000001	1656051	1655950	-	ykkC-yxkD
GCA_021463765.1	Nitrosomonas sp.	AOB - Nitrosomonadaceae	PRJNA791618	JAJTXI010000001	1656279	1656380	+	ykkC-yxkD
GCA_021582655.1	Nitrosomonas sp.	AOB - Nitrosomonadaceae	PRJNA779449	CP091135	1990097	1990202	+	ykkC-yxkD
GCA_021582655.1	Nitrosomonas sp.	AOB - Nitrosomonadaceae	PRJNA779449	CP091135	2088160	2088264	+	ykkC-yxkD
GCA_021604405.1	Nitrosomonas sp.	AOB - Nitrosomonadaceae	PRJDB12579	BQJA01000001	33516	33618	+	ykkC-yxkD
GCA_021604405.1	Nitrosomonas sp.	AOB - Nitrosomonadaceae	PRJDB12579	BQJA01000009	54332	54381	+	mini-ykkC
GCA_021604485.1	Nitrosomonas sp.	AOB - Nitrosomonadaceae	PRJDB12579	BQJB01000014	11026	10980	-	mini-ykkC
GCA_021604485.1	Nitrosomonas sp.	AOB - Nitrosomonadaceae	PRJDB12579	BQJB01000040	8153	8006	-	ykkC-yxkD
GCA_021731985.1	Nitrosomonas sp.	AOB - Nitrosomonadaceae	PRJNA533545	JAKFWH010000084	11227	11122	-	ykkC-yxkD
GCA_021774035.1	Nitrosomonas sp.	AOB - Nitrosomonadaceae	PRJNA755678	JAJFJM010000016	2085	2036	-	mini-ykkC
GCA_021774035.1	Nitrosomonas sp.	AOB - Nitrosomonadaceae	PRJNA755678	JAJFJM010000066	2516	2414	-	ykkC-yxkD
GCA_021774075.1	Nitrosomonas sp.	AOB - Nitrosomonadaceae	PRJNA755678	JAJFJK010000141	29373	29477	+	ykkC-yxkD
GCA_022352095.1	Nitrosomonas sp.	AOB - Nitrosomonadaceae	PRJNA779449	JAKRWR010000076	2394	2499	+	ykkC-yxkD
GCA_022836435.1	Nitrosomonas sp. PY1	AOB - Nitrosomonadaceae	PRJDB5489	BQXC01000001	698678	698792	+	ykkC-yxkD
GCA_022841425.1	Nitrosomonas ureae	AOB - Nitrosomonadaceae	PRJNA764197	JALHRN010000062	8272	8319	+	mini-ykkC
GCA_022841425.1	Nitrosomonas ureae	AOB - Nitrosomonadaceae	PRJNA764197	JALHRN010000141	173	69	-	ykkC-yxkD
GCA_022842015.1	Nitrosomonas sp.	AOB - Nitrosomonadaceae	PRJNA764197	JALHQJ010000002	20928	21032	+	ykkC-yxkD
GCA_022842015.1	Nitrosomonas sp.	AOB - Nitrosomonadaceae	PRJNA764197	JALHQJ010000066	6760	6714	-	mini-ykkC
GCA_023257295.1	Nitrosomonas sp.	AOB - Nitrosomonadaceae	PRJNA730330	JALRCN010000187	7317	7205	-	ykkC-yxkD
GCA_900100485.1	Nitrosomonas sp. Nm132	AOB - Nitrosomonadaceae	PRJEB15933	FNDH01000011	46041	46147	+	ykkC-yxkD
GCA_900100485.1	Nitrosomonas sp. Nm132	AOB - Nitrosomonadaceae	PRJEB15933	FNDH01000077	1068	1022	-	mini-ykkC
GCA_900100815.1	Nitrosomonas eutropha	AOB - Nitrosomonadaceae	PRJEB15888	FMTW01000014	40457	40354	-	ykkC-yxkD
GCA_900100815.1	Nitrosomonas eutropha	AOB - Nitrosomonadaceae	PRJEB15888	FMTW01000014	40670	40771	+	ykkC-yxkD
GCA_900100985.1	Nitrosospira sp. Nsp1	AOB - Nitrosomonadaceae	PRJEB16022	FMUF01000014	51369	51472	+	ykkC-yxkD
GCA_900101945.1	Nitrosospira sp. Nsp13	AOB - Nitrosomonadaceae	PRJEB15791	FMVD01000021	26899	26794	-	ykkC-yxkD
GCA_900102495.1	Nitrosospira sp. Nl5	AOB - Nitrosomonadaceae	PRJEB15853	FMVQ01000025	499	604	+	ykkC-yxkD
GCA_900103035.1	Nitrosomonas mobilis	AOB - Nitrosomonadaceae	PRJEB15545	FMWO01000079	10126	10082	-	mini-ykkC
GCA_900103035.1	Nitrosomonas mobilis	AOB - Nitrosomonadaceae	PRJEB15545	FMWO01000094	26715	26611	-	ykkC-yxkD
GCA_900103145.1	Nitrosospira sp. Nsp18	AOB - Nitrosomonadaceae	PRJEB16264	FMWY01000029	34146	34043	-	ykkC-yxkD
GCA_900103165.1	Nitrosospira multiformis	AOB - Nitrosomonadaceae	PRJEB16165	FNKY01000001	2975657	2975552	-	ykkC-yxkD
GCA_900105875.1	Nitrosomonas ureae	AOB - Nitrosomonadaceae	PRJEB16496	FNLN01000001	166664	166560	-	ykkC-yxkD
GCA_900105875.1	Nitrosomonas ureae	AOB - Nitrosomonadaceae	PRJEB16496	FNLN01000016	58601	58648	+	mini-ykkC
GCA_900106545.1	Nitrosomonas communis	AOB - Nitrosomonadaceae	PRJEB16556	FNNH01000007	40184	40078	-	ykkC-yxkD
GCA_900106555.1	Nitrosomonas oligotropha	AOB - Nitrosomonadaceae	PRJEB16552	FNOE01000006	129695	129800	+	ykkC-yxkD
GCA_900106625.1	Nitrosomonas europaea	AOB - Nitrosomonadaceae	PRJEB16577	FNNX01000029	31856	31753	-	ykkC-yxkD
GCA_900106625.1	Nitrosomonas europaea	AOB - Nitrosomonadaceae	PRJEB16577	FNNX01000029	32074	32176	+	ykkC-yxkD
GCA_900106625.1	Nitrosomonas europaea	AOB - Nitrosomonadaceae	PRJEB16577	FNNX01000058	1669	1719	+	mini-ykkC
GCA_900106875.1	Nitrosomonas eutropha	AOB - Nitrosomonadaceae	PRJEB16565	FNNM01000008	29114	29011	-	ykkC-yxkD
GCA_900106875.1	Nitrosomonas eutropha	AOB - Nitrosomonadaceae	PRJEB16565	FNNM01000008	29327	29427	+	ykkC-yxkD
GCA_900107165.1	Nitrosomonas halophila	AOB - Nitrosomonadaceae	PRJEB16612	FNOY01000003	28964	29008	+	mini-ykkC
GCA_900107165.1	Nitrosomonas halophila	AOB - Nitrosomonadaceae	PRJEB16612	FNOY01000063	13174	13066	-	ykkC-yxkD
GCA_900107265.1	Nitrosomonas sp. Nm33	AOB - Nitrosomonadaceae	PRJEB16537	FNPJ01000048	9161	9054	-	ykkC-yxkD
GCA_900107335.1	Nitrosomonas sp. Nm58	AOB - Nitrosomonadaceae	PRJEB16562	FNPQ01000020	22131	22025	-	ykkC-yxkD
GCA_900107335.1	Nitrosomonas sp. Nm58	AOB - Nitrosomonadaceae	PRJEB16562	FNPQ01000086	4859	4905	+	mini-ykkC
GCA_900107715.1	Nitrosospira multiformis	AOB - Nitrosomonadaceae	PRJEB16654	FNQL01000004	113599	113702	+	ykkC-yxkD
GCA_900108135.1	Nitrosospira multiformis ATCC 25196	AOB - Nitrosomonadaceae	PRJEB16695	FNVK01000002	134714	134817	+	ykkC-yxkD
GCA_900108305.1	Nitrosomonas ureae	AOB - Nitrosomonadaceae	PRJEB16712	FNUX01000002	241222	241118	-	ykkC-yxkD
GCA_900108305.1	Nitrosomonas ureae	AOB - Nitrosomonadaceae	PRJEB16712	FNUX01000034	19064	19114	+	mini-ykkC
GCA_900108975.1	Nitrosomonas eutropha	AOB - Nitrosomonadaceae	PRJEB16773	FNYF01000015	29110	29007	-	ykkC-yxkD
GCA_900108975.1	Nitrosomonas eutropha	AOB - Nitrosomonadaceae	PRJEB16773	FNYF01000015	29323	29423	+	ykkC-yxkD
GCA_900109785.1	Nitrosovibrio tenuis	AOB - Nitrosomonadaceae	PRJEB16870	FOBH01000003	269641	269745	+	ykkC-yxkD
GCA_900110145.1	Nitrosomonas marina	AOB - Nitrosomonadaceae	PRJEB16912	FOCP01000002	182922	182791	-	ykkC-yxkD
GCA_900110145.1	Nitrosomonas marina	AOB - Nitrosomonadaceae	PRJEB16912	FOCP01000009	76253	76211	-	mini-ykkC
GCA_900110185.1	Nitrosospira multiformis	AOB - Nitrosomonadaceae	PRJEB16916	FOCT01000004	124398	124502	+	ykkC-yxkD
GCA_900110385.1	Nitrosomonas oligotropha	AOB - Nitrosomonadaceae	PRJEB16938	FODO01000006	129694	129799	+	ykkC-yxkD
GCA_900110495.1	Nitrosovibrio sp. Nv6	AOB - Nitrosomonadaceae	PRJEB16963	FODX01000003	161572	161454	-	ykkC-yxkD
GCA_900111055.1	Nitrosomonas ureae	AOB - Nitrosomonadaceae	PRJEB17008	FOFX01000011	50815	50919	+	ykkC-yxkD
GCA_900111055.1	Nitrosomonas ureae	AOB - Nitrosomonadaceae	PRJEB17008	FOFX01000016	53279	53326	+	mini-ykkC
GCA_900111165.1	Nitrosomonas sp. Nm51	AOB - Nitrosomonadaceae	PRJEB17019	FOGH01000008	50040	49938	-	ykkC-yxkD
GCA_900111165.1	Nitrosomonas sp. Nm51	AOB - Nitrosomonadaceae	PRJEB17019	FOGH01000024	24880	24838	-	mini-ykkC
GCA_900111165.1	Nitrosomonas sp. Nm51	AOB - Nitrosomonadaceae	PRJEB17019	FOGH01000060	415	558	+	ykkC-yxkD
GCA_900111585.1	Nitrosospira multiformis	AOB - Nitrosomonadaceae	PRJEB17071	FOHI01000005	97758	97862	+	ykkC-yxkD
GCA_900111605.1	Nitrosomonas marina	AOB - Nitrosomonadaceae	PRJEB17062	FOIA01000008	69325	69281	-	mini-ykkC
GCA_900111605.1	Nitrosomonas marina	AOB - Nitrosomonadaceae	PRJEB17062	FOIA01000017	66719	66849	+	ykkC-yxkD
GCA_900111725.1	Nitrosomonas europaea	AOB - Nitrosomonadaceae	PRJEB17031	FOID01000028	31856	31753	-	ykkC-yxkD
GCA_900111725.1	Nitrosomonas europaea	AOB - Nitrosomonadaceae	PRJEB17031	FOID01000028	32074	32176	+	ykkC-yxkD
GCA_900111725.1	Nitrosomonas europaea	AOB - Nitrosomonadaceae	PRJEB17031	FOID01000058	1565	1615	+	mini-ykkC
GCA_900112825.1	Nitrosomonas sp. Nm166	AOB - Nitrosomonadaceae	PRJEB17241	FONE01000005	59366	59320	-	mini-ykkC
GCA_900112825.1	Nitrosomonas sp. Nm166	AOB - Nitrosomonadaceae	PRJEB17241	FONE01000017	48401	48502	+	ykkC-yxkD

GCA_900113575.1	Nitrosospira sp. Nsp14	AOB - Nitrosomonadaceae	PRJEB17340	FOPV01000008	53168	53058	-	ykkC-yxkD
GCA_900113925.1	Nitrosomonas sp. Nm34	AOB - Nitrosomonadaceae	PRJEB17379	FORD01000025	40757	40651	-	ykkC-yxkD
GCA_900114305.1	Nitrosomonas aestuarii	AOB - Nitrosomonadaceae	PRJEB17424	FOSP01000002	41466	41422	-	mini-ykkC
GCA_900114305.1	Nitrosomonas aestuarii	AOB - Nitrosomonadaceae	PRJEB17424	FOSP01000008	93054	92917	-	ykkC-yxkD
GCA_900114745.1	Nitrosomonas communis	AOB - Nitrosomonadaceae	PRJEB17477	FOUB01000030	6707	6813	+	ykkC-yxkD
GCA_900114795.1	Nitrosomonas nitrosa	AOB - Nitrosomonadaceae	PRJEB17482	FOUF01000003	51293	51176	-	ykkC-yxkD
GCA_900115125.1	Nitrosospira briensis	AOB - Nitrosomonadaceae	PRJEB17140	FOVJ01000001	148133	148244	+	ykkC-yxkD
GCA_900115325.1	Nitrosospira briensis	AOB - Nitrosomonadaceae	PRJEB17164	FOVY01000003	109268	109379	+	ykkC-yxkD
GCA_900115725.1	Nitrosomonas cryotolerans	AOB - Nitrosomonadaceae	PRJEB17435	FOXN01000057	9527	9422	-	ykkC-yxkD
GCA_900116685.1	Nitrosomonas eutropha	AOB - Nitrosomonadaceae	PRJEB17588	FPBL01000005	90042	89941	-	ykkC-yxkD
GCA_900116685.1	Nitrosomonas eutropha	AOB - Nitrosomonadaceae	PRJEB17588	FPBL01000005	90340	90443	+	ykkC-yxkD
GCA_900116835.1	Nitrosospira multiformis	AOB - Nitrosomonadaceae	PRJEB17586	FPBZ01000003	160382	160278	-	ykkC-yxkD
GCA_900119085.1	Nitrosovibrio sp. Nv17	AOB - Nitrosomonadaceae	PRJEB17764	FPIQ01000033	6710	6604	-	ykkC-yxkD
GCA_900142705.1	Nitrosospira sp. Nsp11	AOB - Nitrosomonadaceae	PRJEB18380	FRBV01000004	18536	18639	+	ykkC-yxkD
GCA_900143275.1	Nitrosomonas cryotolerans ATCC 49181	AOB - Nitrosomonadaceae	PRJEB18452	FSRO01000001	510230	510335	+	ykkC-yxkD
GCA_900167395.1	Nitrosomonas europaea	AOB - Nitrosomonadaceae	PRJEB19552	FUWK01000029	32058	31955	-	ykkC-yxkD
GCA_900167395.1	Nitrosomonas europaea	AOB - Nitrosomonadaceae	PRJEB19552	FUWK01000029	32276	32378	+	ykkC-yxkD
GCA_900167395.1	Nitrosomonas europaea	AOB - Nitrosomonadaceae	PRJEB19552	FUWK01000057	11216	11166	-	mini-ykkC
GCA_900206265.1	Nitrosomonas ureae	AOB - Nitrosomonadaceae	PRJEB22288	LT907782	2053166	2053270	+	ykkC-yxkD
GCA_900206265.1	Nitrosomonas ureae	AOB - Nitrosomonadaceae	PRJEB22288	LT907782	2549907	2549954	+	mini-ykkC
GCA_900215345.1	Nitrosomonas ureae	AOB - Nitrosomonadaceae	PRJEB22428	OCMU01000001	1700384	1700280	-	ykkC-yxkD
GCA_900215345.1	Nitrosomonas ureae	AOB - Nitrosomonadaceae	PRJEB22428	OCMU01000001	2022750	2022797	+	mini-ykkC
GCA_900215485.1	Nitrosovibrio sp. Nv4	AOB - Nitrosomonadaceae	PRJEB22441	OCMZ01000001	1512536	1512654	+	ykkC-yxkD
GCA_902826115.1	uncultured Nitrosomonas sp.	AOB - Nitrosomonadaceae	PRJEB30654	CADEDO010000009	32396	32500	+	ykkC-yxkD
GCA_902826915.1	uncultured Nitrosomonas sp.	AOB - Nitrosomonadaceae	PRJEB30654	CADEGP010000003	53314	53418	+	ykkC-yxkD
GCA_905220645.1	Nitrosomonas nitrosa	AOB - Nitrosomonadaceae	PRJEB34639	CAJNAP010000001	135298	135181	-	ykkC-yxkD
GCA_919902625.1	Nitrosomonadaceae bacterium	AOB - Nitrosomonadaceae	PRJEB36523	CAKKRO010000136	6338	6233	-	ykkC-yxkD
GCA_919902635.1	Nitrosomonadaceae bacterium	AOB - Nitrosomonadaceae	PRJEB36523	CAKKRR010000521	2141	2036	-	ykkC-yxkD
GCA_000024725.1	Nitrosococcus halophilus Nc 4	AOB - Nitrosococcaceae	PRJNA36589	CP001798	2696642	2696692	+	mini-ykkC
GCA_000200715.1	Cenarchaeum symbiosum A	AOA	PRJNA202	DP000238	92456	92406	-	mini-ykkC
GCA_009836545.1	Cenarchaeum sp. SB0667_bin_13	AOA	PRJNA555144	VXVG01000002	4031	3973	-	mini-ykkC
GCA_900620265.1	Nitrosopumilaceae archaeon	AOA	PRJEB29556	UYNY01000031	10104	10054	-	mini-ykkC
GCA_900620265.1	Nitrosopumilaceae archaeon	AOA	PRJEB29556	UYNY01000043	4138	4068	-	mini-ykkC

Supplementary Table 3. Counts of guanidine riboswitches and guanidine degradation genes in genomes of comammox *Nitrospira*. Counts for guanidine degradation genes that are immediately downstream from guanidine riboswitches are differentiated from counts for those genes that are not found immediately downstream from a guanidine riboswitch.

			Riboswitches			Counts of genes downstream from guanidine-I riboswitch						Counts of genes not downstream from identifiable riboswitch					
Genbank assembly accession	Genbank Name	Type	Guanidine I (ykkC-	miniykkC	ykkCIII	AA permease	Guanidine Carboxyla	CgdA	CgdB	Allophanate	Guanidinase	AA permease	Guanidine Carboxyla	CgdA	CgdB	Allophanate	Guanidinase
GCA_001458695	*Nitrospira inopinata*	Comammox clade A	1	0	0	1	0	0	0	0	1	0	0	0	0	0	0
GCA_001458735	*Candidatus* Nitrospira nitrosa	Comammox clade A	1	0	0	1	0	0	0	0	1	0	0	0	0	0	0
GCA_001458775	*Candidatus* Nitrospira nitrificans	Comammox clade A	1	0	1	1	0	0	0	0	1	0	0	0	0	0	0
GCA_014058405	*Candidatus* Nitrospira kreftii	Comammox clade A	1	0	0	1	0	0	0	0	1	0	0	0	0	0	0
GCA_016722055	Nitrospira sp. (Ribe MAG)	Comammox clade A	1	0	0	1	0	0	0	0	1	0	0	0	0	0	0
GCA_016712165	Nitrospira sp. (Haderslev MAG)	Comammox clade A	1	0	0	1	0	0	0	0	1	0	0	0	0	0	0
GCA_001464735	Nitrospira sp. Ga0074138	Comammox clade A	2	0	0	1	0	0	0	0	0	1	0	0	0	0	2
GCA_002083355	Nitrospira sp. HN-bin3	Comammox clade A	0	0	0	0	0	0	0	0	0	0	0	0	0	0	1
GCA_002083365	Nitrospira sp. SG-bin1	Comammox clade A	1	0	1	1	0	0	0	0	1	0	0	0	0	0	0
GCA_002083405	Nitrospira sp. SG-bin2	Comammox clade A	1	0	0	1	0	0	0	0	1	0	0	0	0	0	0
GCA_002083565	Nitrospira sp. ST-bin4	Comammox clade A	1	0	0	1	0	0	0	0	0	0	0	0	0	0	0
GCA_002254365	Nitrospira sp. UW-LDO-01	Comammox clade A	1	0	0	1	0	0	0	0	1	0	0	0	0	0	0
GCA_002331335	Nitrospira sp. UBA2082	Comammox clade A	1	1	0	1	0	0	0	0	1	0	0	0	0	0	0
GCA_002331625	Nitrospira sp. UBA2083	Comammox clade A	0	0	0	0	0	0	0	0	0	1	0	0	0	0	0
GCA_002420045	Nitrospira sp. UBA5702	Comammox clade A	1	0	0	2	0	0	0	0	1	0	0	0	0	0	0
GCA_002869845	Nitrospira sp. CG24B	Comammox clade A	0	0	0	0	0	0	0	0	0	0	0	0	0	0	0
GCA_003529185	Nitrospira sp.	Comammox clade A	1	0	0	1	0	0	0	0	1	0	0	0	0	0	0
GCA_005116745	Nitrospira sp.	Comammox clade A	1	1	0	1	0	0	0	0	1	0	0	0	0	0	0
GCA_005116895	Nitrospira sp.	Comammox clade A	1	1	0	1	0	0	0	0	1	0	0	0	0	0	0
GCA_005116955	Nitrospira sp.	Comammox clade A	1	0	0	1	0	0	0	0	1	0	0	0	0	0	0
GCA_005116965	Nitrospira sp.	Comammox clade A	1	0	0	1	0	0	0	0	1	0	0	0	0	0	0
GCA_005239465	Nitrospira sp.	Comammox clade A	0	0	0	0	0	0	0	0	0	0	0	0	0	1	0
GCA_009594825	Nitrospira sp.	Comammox clade A	1	0	0	1	0	0	0	0	1	0	0	0	0	0	0
GCA_009594935	Nitrospira sp.	Comammox clade A	0	0	0	0	0	0	0	0	0	0	0	0	0	0	0
GCA_009595005	Nitrospira sp.	Comammox clade A	1	0	0	1	0	0	0	0	1	0	0	0	0	0	0
GCA_011090395	Nitrospira sp. LK265	Comammox clade A	1	0	0	1	0	0	0	0	1	0	0	0	0	0	0
GCA_011090425	Nitrospira sp. LK70	Comammox clade A	1	0	0	1	0	0	0	0	1	0	0	0	0	0	0
GCA_012032965	Nitrospira sp.	Comammox clade A	1	0	0	1	0	0	0	0	0	1	0	0	0	0	1
GCA_013141205	Nitrospira sp.	Comammox clade A	1	0	0	1	0	0	0	0	1	0	0	0	0	0	0
GCA_015903945	Nitrospira sp.	Comammox clade A	1	0	0	1	0	0	0	0	1	0	0	0	0	0	0
GCA_015903955	Nitrospira sp.	Comammox clade A	1	0	0	1	0	0	0	0	1	0	0	0	0	0	0
GCA_015903965	Nitrospira sp.	Comammox clade A	0	0	0	0	0	0	0	0	0	1	0	0	0	0	1
GCA_015903995	Nitrospira sp.	Comammox clade A	1	0	0	1	0	0	0	0	1	1	0	0	0	0	1
GCA_015904045	Nitrospira sp.	Comammox clade A	2	0	0	1	0	0	0	0	1	0	0	0	0	0	0
GCA_016222925	Nitrospirae bacterium	Comammox clade A	0	0	0	0	0	0	0	0	0	0	0	0	0	0	1
GCA_016715825	Nitrospira sp.	Comammox clade A	1	0	0	1	0	0	0	0	1	0	0	0	0	0	0
GCA_016788465	Nitrospira sp.	Comammox clade A	1	0	0	1	0	0	0	0	1	0	0	0	0	0	0
GCA_016789065	Nitrospira sp.	Comammox clade A	1	0	0	1	0	0	0	0	1	0	0	0	0	0	0
GCA_016873455	Nitrospira sp.	Comammox clade A	1	0	0	1	0	0	0	0	1	0	0	0	0	0	0
GCA_016873515	Nitrospira sp.	Comammox clade A	0	0	0	0	0	0	0	0	0	0	0	0	0	0	0
GCA_018242685	Nitrospira sp.	Comammox clade A	1	0	0	1	0	0	0	0	1	0	0	0	0	0	0
GCA_018242765	Nitrospira sp.	Comammox clade A	1	0	0	1	0	0	0	0	1	0	0	0	0	0	0
GCA_018242825	Nitrospira sp.	Comammox clade A	1	0	0	1	0	0	0	0	1	0	0	0	0	0	0
GCA_019636815	Nitrospira sp.	Comammox clade A	1	0	0	1	0	0	0	0	1	0	0	0	0	0	0
GCA_019636835	Nitrospira sp.	Comammox clade A	1	0	0	1	0	0	0	0	1	0	0	0	0	0	0
GCA_019636875	Nitrospira sp.	Comammox clade A	0	0	0	0	0	0	0	0	0	1	0	0	0	0	1
GCA_019636915	Nitrospira sp.	Comammox clade A	1	0	0	1	0	0	0	0	0	1	0	0	0	0	1
GCA_019636945	Nitrospira sp.	Comammox clade A	1	0	0	1	0	0	0	0	1	0	0	0	0	0	0
GCA_019750685	Nitrospiraceae bacterium	Comammox clade A	1	0	0	1	0	0	0	0	1	0	0	0	0	0	0
GCA_020216985	Nitrospira sp.	Comammox clade A	0	0	0	0	0	0	0	0	0	0	0	0	0	0	0
GCA_022842445	Nitrospira sp.	Comammox clade A	1	0	0	1	0	0	0	0	1	0	0	0	0	0	0
GCA_022843765	Nitrospira sp.	Comammox clade A	0	0	0	0	0	0	0	0	0	0	0	0	0	0	0
GCA_022843785	Nitrospira sp.	Comammox clade A	1	0	0	1	0	0	0	0	1	0	0	0	0	0	0
GCA_900696515	uncultured Nitrosospira sp.	Comammox clade A	1	0	0	1	0	0	0	0	1	0	0	0	0	0	0
GCA_902500705	uncultured Nitrospira sp.	Comammox clade A	1	1	0	1	0	0	0	0	0	0	0	0	0	0	0
GCA_902500745	uncultured Nitrospira sp.	Comammox clade A	1	0	0	1	0	0	0	0	0	0	0	0	0	0	0
GCA_902500755	uncultured Nitrospira sp.	Comammox clade A	4	0	0	1	0	0	0	0	0	0	0	0	0	0	1
GCA_902500775	uncultured Nitrospira sp.	Comammox clade A	1	0	0	1	0	0	0	0	1	0	0	0	0	0	0
GCA_902500795	uncultured Nitrospira sp.	Comammox clade A	2	0	0	1	0	0	0	0	0	0	0	0	0	0	0
GCA_902500805	uncultured Nitrospira sp.	Comammox clade A	1	0	0	1	0	0	0	0	1	0	0	0	0	0	0
GCA_902500825	uncultured Nitrospira sp.	Comammox clade A	1	0	0	1	0	0	0	0	1	0	0	0	0	0	0
GCA_902501035	uncultured Nitrospira sp.	Comammox clade A	1	0	0	1	0	0	0	0	1	0	0	0	0	0	0
GCA_919902665	Nitrospiraceae bacterium	Comammox clade A	1	0	0	1	0	0	0	0	1	0	0	0	0	0	0
GCA_002869885	Nitrospira sp. CG24C	Comammox clade B	0	0	0	0	0	0	0	0	0	1	0	0	0	0	1
GCA_002869895	Nitrospira sp. CG24E	Comammox clade B	0	0	0	0	0	0	0	0	0	1	0	0	0	0	1
GCA_002869925	Nitrospira sp. CG24A	Comammox clade B	0	0	0	0	0	0	0	0	0	1	0	0	0	0	1
GCA_003135435	Nitrospira sp.	Comammox clade B	0	0	0	0	0	0	0	0	0	1	1	1	1	0	0
GCA_005116775	Nitrospira sp.	Comammox clade B	0	0	0	0	0	0	0	0	0	1	0	0	0	0	0
GCA_005116825	Nitrospira sp.	Comammox clade B	0	0	0	0	0	0	0	0	0	1	0	0	0	0	1
GCA_005116835	Nitrospira sp.	Comammox clade B	0	0	0	0	0	0	0	0	0	1	0	0	0	0	1
GCA_005116885	Nitrospira sp.	Comammox clade B	0	0	0	0	0	0	0	0	0	0	0	0	0	0	0
GCA_005239475	Nitrospira sp.	Comammox clade B	2	0	0	2	0	0	0	0	2	0	0	0	0	0	0
GCA_005887945	Nitrospirae bacterium	Comammox clade B	0	0	0	0	0	0	0	0	0	1	0	0	0	0	1
GCA_011391455	Nitrospiraceae bacterium	Comammox clade B	0	0	0	0	0	0	0	0	0	0	0	0	0	0	1
GCA_013140535	Nitrospira sp.	Comammox clade B	0	0	0	0	0	0	0	0	0	1	0	0	0	0	1
GCA_013140715	Nitrospiraceae bacterium	Comammox clade B	1	0	0	1	1	1	1	0	0	0	0	0	0	0	0
GCA_013388555	Nitrospirae bacterium	Comammox clade B	0	0	0	0	0	0	0	0	0	1	0	0	0	0	0
GCA_014379455	Nitrospiraceae bacterium	Comammox clade B	0	0	0	0	0	0	0	0	0	0	0	0	0	0	0
GCA_016200355	Nitrospirae bacterium	Comammox clade B	0	0	0	0	0	0	0	0	0	1	0	0	0	0	0
GCA_016219645	Nitrospirae bacterium	Comammox clade B	0	1	0	0	0	0	0	0	0	1	0	0	0	0	1
GCA_016880205	Nitrospira sp.	Comammox clade B	0	0	0	0	0	0	0	0	0	1	0	0	0	0	1
GCA_919901595	Nitrospiraceae bacterium	Comammox clade B	1	0	0	1	0	0	0	0	1	0	0	0	0	0	0
GCA_919902055	Nitrospira bact. HGW-Nitrospira-1	Comammox clade B	1	0	0	1	0	0	0	0	0	0	0	0	0	0	0

Supplementary Table 4. Amino acids potentially involved in metal binding and catalysis of guanidine. The function and identity of the amino acid are given as well as the location in *N. inopinata* (CUQ66148.1), the crystal structure of Sll1077 from *Synechocystis* sp. PCC 6803 (P73270.1) and the PFAM model for ureohydrolase (PF00491)

Function	AA	CUQ66148.1 (*N. inopinata*)	P73270.1 [17,18]	PFAM model PF00491.24
metal binding	H	182	174	93
metal binding	D	207	199	117
metal binding	H	209	201	119
metal binding	D	211	203	121
metal binding	D	299	291	209
metal binding	D	301	293	211
substrate catalysis	T	105	97	15
substrate catalysis	H	222	214	134
substrate catalysis	W	313	305	223
substrate catalysis	E	344	338	254

Supplementary Table 5. Peak areas of products after derivatization of guanidine. Peak areas of products c and d (see Supplementary Figure 2) after derivatization of guanidine and arginine in MilliQ water with ethanol or 2-methoxyethanol as co-solvent. Arginine (2-amino-5-guanidinopentanoic acid) was used as a biologically relevant control for substituted guanidino compounds, which can result in the same derivatization product as guanidine. Reported values represent means ± standard deviations of triplicate derivatizations.

Reactant	Solvent	Peak area of product 3$^{(*)}$ m/z: 252.2, (x10^6)	Peak area of product 4$^{(*)}$ m/z: 236.2, (x10^6)
Guanidine (100 µM)	2-Methoxyethanol	183 ± 7	17.5 ± 1.1
Guanidine (100 µM)	Ethanol	266 ± 5	0.71 ± 0.14
Blank (0 µM)	Ethanol	0.54 ± 0.03	0.040 ± 0.017
Arginine (100 µM)	Ethanol	7.7 ± 0.8	0.15 ± 0.05

Supplementary Table 6. Chemical analyses, cell counts, rate and yields from *N. inopinata* physiology experiment. This table is provided as supplementary information as separate file in .xlsx (MS Excel) format.

Supplementary Table 7. Interactions of the N-terminal extension of the guanidinase of N. inopinata with its own subunit. Columns A-C denote the residue interaction with the N-terminal extension, columns D-E denote residue of the N-terminal extension involved in the interaction, columns F-I describe interaction. Distance H-A [Å]: Hydrogen - Acceptor distance, Distance D-A [Å]: Donor - Acceptor distance, Distance A-W [Å]: Acceptor - Water distance, Distance D-W [Å]: Donor - Water distance. Interactions were calculated using PLIP [97].

Hydrogen bonds

Nr	Residue	Chain	Nr N-Term	Residue N-Term	Distance H-A [Å]	Distance D-A [Å]	Donor angle [°]
21	ALA	C	18	GLY	2.81	3.18	107.87
22	LYS	C	18	GLY	1.97	2.75	151.01
26	GLU	C	9	GLY	3.56	4.07	121.5
26	GLU	C	8	GLN	1.92	2.74	159.32
26	GLU	C	11	VAL	2.25	3.08	163.07
26	GLU	C	10	LYS	2.15	2.77	128.54
263	THR	F	14	HIS	1.98	2.83	168.48
265	ILE	F	18	GLY	2.26	3.09	161.54
282	GLN	F	16	ASN	2.26	2.84	124.96
372	ARG	C	5	ARG	3.38	4	131.42
372	ARG	C	5	ARG	2.49	3.28	153.05

Water Bridges

Nr	Residue	Chain	Nr N-Term	Residue N-Term	Distance A-W [Å]	Distance D-W [Å]	Donor angle [°]	Water angle [°]
23	TYR	C	7	TYR	2.86	3.95	125.24	96.1
258	GLY	F	17	TYR	3.22	2.57	173.19	86.21
262	GLN	F	14	HIS	3.99	3.9	111.48	79.14
262	GLN	F	17	TYR	2.72	3.9	111.48	77.23
262	GLN	F	14	HIS	2.91	3.38	153.75	74.6
263	THR	F	17	TYR	3.41	2.57	173.19	113.35
264	THR	F	14	HIS	4.01	3.77	114.56	109.09

Salt Bridges

Nr	Residue	Chain	Nr N-Term	Residue N-Term	Distance [Å]	Protein positively charged	Interacting group
22	LYS	C	15	ASP	4.87	True	Carboxylate

Hydrophobic interactions

Nr	Residue	Chain	Nr N-Term	Residue N-Term	Distance [Å]
22	LYS	C	17	TYR	3.75
22	LYS	C	7	TYR	3.94
22	LYS	C	7	TYR	3.62
23	TYR	C	5	ARG	3.83
25	VAL	C	17	TYR	3.81
26	GLU	C	11	VAL	3.81
29	ALA	C	11	VAL	3.93
30	LEU	C	11	VAL	3.89

Supplementary Table 8. Interactions of the C-terminal extension of the guanidinase of *Synechocystis* (GdmH) with its own subunit. Columns A-C denote the residue interaction with the C-terminal extension, columns D-E denote residue of the C-terminal extension involved in the interaction, columns F-I describe interaction. Distance H-A [Å]: Hydrogen - Acceptor distance, Distance D-A [Å]: Donor - Acceptor distance, Distance A-W [Å]: Acceptor - Water distance, Distance D-W [Å]: Donor - Water distance. Interactions were calculated using PLIP [97].

Hydrogen bonds

Nr	Residue	Chain	Nr C-Term	Residue C-Term	Distance H-A [Å]	Distance D-A [Å]	Donor angle [°]
162	SER	A	373	LYS	3.41	4.07	123.84
164	GLY	A	370	ARG	2.67	3.29	121.16
182	ARG	A	386	ASP	1.83	2.78	161.36
186	ARG	A	382	ASN	1.88	2.76	147.97
187	HIS	A	378	HIS	1.74	2.71	169.81
187	HIS	A	378	HIS	1.99	2.85	145.25
328	ASN	A	376	TYR	3.4	3.92	114.59
328	ASN	A	376	TYR	2.5	3.38	157.13
329	VAL	A	370	ARG	3.42	3.94	114.51

Water Bridges

Nr	Residue	Chain	Nr C-Term	Residue C-Term	Distance A-W [Å]	Distance D-W [Å]	Donor angle [°]	Water angle [°]
84	ASP	A	370	ARG	3.57	2.82	124.41	72.05
166	PHE	A	370	ARG	3.81	3.39	113.81	86.35
190	ASP	A	378	HIS	2.56	3.42	157.51	82.19
212	ARG	A	386	ASP	2.53	3.25	144.94	71.72

Hydrophobic interactions

Nr	Residue	Chain	Nr C-Term	Residue C-Term	Distance [Å]
161	PHE	A	376	TYR	3.87
182	ARG	A	385	VAL	3.99
186	ARG	A	385	VAL	3.5
191	LYS	A	376	TYR	3.61

Supplementary Table 9. Summary of distances of nickel and manganese ions to their respective interacting residue. Distance statistics are calculated over all 12 subunits in the asymmetric unit.

	Mean distance [Å]	Standard deviation	Minimum distance [Å]	25%	50%	75%	Maximum distance [Å]
**Asp207 – Mn**	1.865	0.042	1.768	1.840	1.872	1.893	1.913
**His209 – Mn**	2.523	0.221	1.963	2.459	2.535	2.622	2.857
**Asp299 – Mn**	2.186	0.162	1.966	2.044	2.160	2.320	2.481
**Asp301 – Mn**	1.725	0.124	1.465	1.689	1.717	1.760	1.970
**His182 – Ni**	2.504	0.236	2.112	2.381	2.482	2.589	3.012
**Asp207 – Ni**	1.865	0.042	1.768	1.840	1.872	1.893	1.913
**Asp211 – Ni**	2.471	0.153	2.158	2.427	2.529	2.555	2.642
**Asp299 – Ni**	2.650	0.122	2.402	2.600	2.646	2.749	2.824
**Mn – Ni**	2.842	0.247	2.472	2.646	2.809	2.980	3.290

Supplementary Table 10. Quantification of *amoA* gene copies (gene copies g^{-1} DW soil) of different ammonia oxidizer clades in ambient (0 days) and incubated (27 days) soil. Values are means and standard deviations (n=3). qPCRs were conducted for AOB, AOA and comammox clade A and B according to Rotthauwe et al., 1997, Alves et al., 2013 and Pjevac et al., 2017 [39,92,93]. No significant differences between treatments or compared to ambient (0 days) were found ($p>0.05$). Statistical differences were tested using a 2-way ANOVA with Tukey's HSD post hoc test with all pairwise comparisons.

Treatment	Inhibitor	AOB mean	AOB STDEV	AOA mean	AOA STDEV	comB mean	comB STDEV	comA mean	comA STDEV
Ambient (0 days)	None	14685246.8	2119311.1	629759.2	103654.9	352796.2	14766.3	106189.2	10450.6
No Nitrogen	None	17617419.0	2526197.7	773032.0	113896.0	421479.8	79555.6	98899.7	7710.9
Guanidine	None	14660013.9	1936118.8	718754.8	47339.5	394182.9	100630.3	81089.5	44131.0
Ammonium	None	20949120.7	3539075.9	1012234.9	323930.0	552298.2	118598.6	94226.9	13441.0
Guanidine + Ammonium	None	21254415.9	4515427.4	956177.5	205519.4	529339.7	48732.1	104901.4	30794.9
No Nitrogen	Acetylene	16768132.6	3044633.7	633558.4	100904.9	357216.6	85412.2	98329.2	38598.3
Guanidine	Acetylene	25263396.1	3509992.9	1202510.6	377041.1	512592.6	94584.7	136124.5	32701.4
Ammonium	Acetylene	18509261.3	1597967.3	1202510.6	61102.9	626397.9	35641.7	92474.6	2154.2
Guanidine + Ammonium	Acetylene	17037803.6	3836234.2	855173.0	267582.8	525312.9	54633.2	79190.2	17235.0

Supplementary Table 11. Methodological details regarding HMMsearch and HMMscan parameters used for the collection of each gene set related to guanidine degradation.

Protein	collection HMM	collection (HMMsearch) parameters	cross check HMMs	cross check (HMMscan) parameters	e-value cutoff	min coverage length (AA)	centroid clustering identity	centroid clustering identity
amino acid/ polyamine/ organocation permease (APC superfamily)	PTHR45649	"-E 0.001 --incE 0.001 --incdomE 0.001 --domE 0.001"	PTHR45649, TIGR03428, PTHR42770	"-E 0.001 --incE 0.001 --incdomE 0.001 --domE 0.001"	1.00E-10	140	0.8	0.7
guanidine carboxylase	TIGR02712	"-E 0.001 --incE 0.001 --incdomE 0.001 --domE 0.001"	TIGR02712, PTHR18866	"-E 0.001 --incE 0.001 --incdomE 0.001 --domE 0.001"	1.00E-03	500	0.8	0.7
carboxyguanidine deiminase (A and B)	PF09347	"-E 0.001 --incE 0.001 --incdomE 0.001 --domE 0.001"	TIGR03424.1, TIGR03425.1, PF09347.13, PTHR31527	"-E 0.001 --incE 0.001 --incdomE 0.001 --domE 0.001"	1.00E-03	150	0.8	0.7
Allophanate hydrolase	TIGR02713	"-E 0.001 --incE 0.001 --incdomE 0.001 --domE 0.001"	TIGR02713, PTHR11895, NF006043	"-E 0.001 --incE 0.001 --incdomE 0.001 --domE 0.001"	1.00E-03	500	0.8	0.7
ureohydrolase (general)	PF00491	"-E 0.001 --incE 0.001 --incdomE 0.001 --domE 0.001"	NF002564, NF010347, PF00491, PTHR11358, PTHR43782, TIGR01227, TIGR01229, TIGR01230	"-E 0.001 --incE 0.001 --incdomE 0.001 --domE 0.001"	1.00E-40	250	0.7	0.7
urease subunit A (ureA)	PF00547	"-E 0.001 --incE 0.001 --incdomE 0.001 --domE 0.001"	PF00547, NF009712, TIGR00193, NF009671, PTHR43440, PTHR33569	"-E 0.001 --incE 0.001 --incdomE 0.001 --domE 0.001"	1.00E-03	85	0.8	0.7
urease subunit B (ureB)	PF00699	"-E 0.001 --incE 0.001 --incdomE 0.001 --domE 0.001"	PF00699, NF009682, PF00699, NF009681, TIGR00192, NF009671, NF010592, PTHR33569, PTHR43440	"-E 0.001 --incE 0.001 --incdomE 0.001 --domE 0.001"	1.00E-03	75	0.8	0.7
urease subunit C (ureC)	PTHR43440	"-E 0.001 --incE 0.001 --incdomE 0.001 --domE 0.001"	NF009686, PTHR43440, TIGR01792, NF009685, NF009834, NF010591, PTHR33569, PF00449, PF01979	"-E 0.001 --incE 0.001 --incdomE 0.001 --domE 0.001"	1.00E-50	400	0.8	0.7 icp

Supplementary Table 12. Data collection and refinement statistics for the *N. inopinata* guanidinase crystal structure. Values in parentheses are for highest-resolution shell.

	Guanidinase	Anomalous Ni	Anomalous Mn
**Data collection**			
Wavelength [Å]	0.9184	1.483	1.893
Space group	P 1 21 1	P 1 21 1	P 1 21 1
Cell dimensions			
a, *b*, *c* (Å)	98.97 164.79 143.97	98.95 164.90 144.01	99.19 165.33 143.87
α, β, γ (°)	90.00, 90.03, 90.00	90.00, 90.04, 90.00	90.00, 90.03, 90.00
Resolution (Å)	45.01 - 1.58 (1.64 - 1.58)	48 - 2.2 (2.23 - 2.2)	47.62 - 2.7 (2.73 - 2.7)
No. reflections total	1339971 (114119)	2118650 (74674)	1088885 (37663)
No. reflections unique	583936 (53686)	586205 (20432)	299828 (10377
R_{sym} or R_{merge}	0.1064 (1.191)	0.1065 (0.6593)	0.1487 (0.5616)
R_{meas}	0.1318 (1.506)	0.2037 (1.268)	0.2856 (1.082)
CC1/2	0.991 (0.256)	0.991 (0.473)	0.977 (0.588)
$I / \sigma I$	4.66 (0.56)	5.05 (1.07)	3.49 (0.89)
Completeness (%)	92.89 (85.24)	95.86 (94.66)	95.26 (94.07)
Redundancy	2.3 (2.1)	3.6 (3.7)	3.6 (3.6)
Wilson B-factor	22.07	23.37	22.07
**Refinement**			
Resolution (Å)	1.58	2.2	2.7
No. reflections	583182 (53352)	224050 (7796)	121217 (4278)
R_{work} / R_{free}	0.2001 / 0.2131	0.1693 / 0.2048	0.1932 / 0.2854
No. atoms	37399	37399	37399
Protein	34440	34440	34440
Ligand/ion	144	24	24
Water	2815	2815	2815
B-factors			
Protein	24.72	25.64	30.55
Ligand/ion	37.52	33.52	36.78
Water	31.51	30.56	33.35
R.m.s. deviations			
Bond lengths (Å)	0.009	0.005	0.012
Bond angles (°)	1.10	0.80	1.31
Ramachandran favored (%)	97.29	97.40	94.11
Ramachandran allowed (%)	2.44	2.31	5.26
Ramachandran outliers (%)	0.27	0.29	0.63
Rotamer outliers (%)	0.14	0.31	1.58
Clashscore	3.33	3.40	11.40

Supplementary References

96. Fan, R.-J. *et al.* Benzylic rearrangement stable isotope labeling for quantitation of guanidino and ureido compounds in thyroid tissues by liquid chromatography electrospray ionization mass spectrometry. *Anal. Chim. Acta* **908**, 132–140 (2016).

97. Adasme, M. F. *et al.* PLIP 2021: expanding the scope of the protein-ligand interaction profiler to DNA and RNA. *Nucleic Acids Res.* **49**, W530–W534 (2021).
