# Supplementary material for: Growth of complete ammonia oxidizers on guanidine
Source: Nature. 2024 Aug 14;633(8030):646–53. doi: 10.1038/s41586-024-07832-z (PMC11410670; doi:10.1038/s41586-024-07832-z)
Supplement: Supplementary file 2 — Reporting Summary [file 41586_2024_7832_MOESM2_ESM.pdf]

nature portfolio

Corresponding author(s): Michael Wagner

Last updated by author(s): Jul 12, 2024

Reporting Summary

Nature Portfolio wishes to improve the reproducibility of the work that we publish. This form provides structure for consistency and transparency in reporting. For further information on Nature Portfolio policies, see our Editorial Policies and the Editorial Policy Checklist.

Statistics

For all statistical analyses, confirm that the following items are present in the figure legend, table legend, main text, or Methods section.

n/a	Confirmed	
☐	☒	The exact sample size (*n*) for each experimental group/condition, given as a discrete number and unit of measurement
☐	☒	A statement on whether measurements were taken from distinct samples or whether the same sample was measured repeatedly
☐	☒	The statistical test(s) used AND whether they are one- or two-sided<br>*Only common tests should be described solely by name; describe more complex techniques in the Methods section.*
☐	☒	A description of all covariates tested
☐	☒	A description of any assumptions or corrections, such as tests of normality and adjustment for multiple comparisons
☐	☒	A full description of the statistical parameters including central tendency (e.g. means) or other basic estimates (e.g. regression coefficient) AND variation (e.g. standard deviation) or associated estimates of uncertainty (e.g. confidence intervals)
☐	☒	For null hypothesis testing, the test statistic (e.g. *F*, *t*, *r*) with confidence intervals, effect sizes, degrees of freedom and *P* value noted<br>*Give P values as exact values whenever suitable.*
☒	☐	For Bayesian analysis, information on the choice of priors and Markov chain Monte Carlo settings
☒	☐	For hierarchical and complex designs, identification of the appropriate level for tests and full reporting of outcomes
☒	☐	Estimates of effect sizes (e.g. Cohen's *d*, Pearson's *r*), indicating how they were calculated

Our web collection on statistics for biologists contains articles on many of the points above.

Software and code

Policy information about availability of computer code

Data collection	hmmsearch, infernal v1.1.3, XDS, SensorTrace Rate, bbmap v38.92, C1000-CFX96 BioRad, NovaSeq 6000
Data analysis	SigmaPlot v14.5, MS Excel 2016, IQ-TREE2, Proteome Discoverer v.2.5, Astra, MoltenProt, MaxEnt1, XDSCONV, Phaser-MR, AlphaFold, COOT, PHENIX, MolProbity, PDBREDO, PyMol v 2.0, DESeq2, R (packages: DEqMS, phangorn, ape, ggtree, ggseqlogo, Decipher,phytools), usearch, FastTree2, OpenMIMS v 3.0.5, Fiji ImageJ 1.54f, bbmap v38.92, bbduk, bedtools

For manuscripts utilizing custom algorithms or software that are central to the research but not yet described in published literature, software must be made available to editors and reviewers. We strongly encourage code deposition in a community repository (e.g. GitHub). See the Nature Portfolio guidelines for submitting code & software for further information.

Data

Policy information about availability of data

All manuscripts must include a data availability statement. This statement should provide the following information, where applicable:

- Accession codes, unique identifiers, or web links for publicly available datasets
- A description of any restrictions on data availability
- For clinical datasets or third party data, please ensure that the statement adheres to our policy

The crystal structures have been deposited in the Protein Data Bank under Code: 9FEK. WWTP metatranscriptome reads have been deposited under BioProject ID PRJNA1118285. The mass spectrometry proteomics data have been deposited to the ProteomeXchange Consortium via the PRIDE 94 partner repository with the

dataset identifier PXD038826. A high-resolution version of Extended Data Figure 4a is deposited under https://doi.org/10.6084/m9.figshare.26139127. Source data are provided with this paper.

Research involving human participants, their data, or biological material

Policy information about studies with human participants or human data. See also policy information about sex, gender (identity/presentation), and sexual orientation and race, ethnicity and racism.

Reporting on sex and gender	Not applicable. No human participants were used in this study.
Reporting on race, ethnicity, or other socially relevant groupings	Not applicable. No human participants were used in this study.
Population characteristics	Not applicable. No human participants were used in this study.
Recruitment	Not applicable. No human participants were used in this study.
Ethics oversight	Not applicable. No human participants were used in this study.

Note that full information on the approval of the study protocol must also be provided in the manuscript.

Field-specific reporting

Please select the one below that is the best fit for your research. If you are not sure, read the appropriate sections before making your selection.

☐ Life sciences ☐ Behavioural & social sciences ☒ Ecological, evolutionary & environmental sciences

For a reference copy of the document with all sections, see nature.com/documents/nr-reporting-summary-flat.pdf

Ecological, evolutionary & environmental sciences study design

All studies must disclose on these points even when the disclosure is negative.

Study description	Ability of Nitrospira inopinata to grow on guanidine as the sole source of energy, reductant, and nitrogen was tested. Proteomics, enzyme kinetics, and the crystal structure of a N. inopinata guanidinase homologue was determined. Transcription of comammox guanidinases was induced in wastewater treatment plant microbiomes upon incubation with guanidine and the nitrifying community which also included comammox, present in natural soil used guanidine as a substrate.
Research sample	Nitrospira inopinata (complete ammonia-oxidizer) bacterial pure culture. Heterologously expressed guanidinase homologue from N.inopinata. Activated sludge samples from wastewater treatment plants. Agricultural soil. Dropped animal manure and urine from cows, pigs, sheep and chicken.
Sampling strategy	For physiological experiments 3 - 5 biological replicates were performed. For shotgun proteomics, 5 biological replicates were performed.
Data collection	Data was collected by small batch culturing of bacteria, chemical analysis, qPCR, shotgun proteomics, chrystal structure analysis, ICP-MS and metatranscriptomics by Christopher J. Sedlacek, Kenneth Wasmund, Nico Jehmlich, Richard Gruseck, Julius Kostan, Dominic Pühringer, Katharina Kitzinger, Andrew Giguere and Marton Palatinszky
Timing and spatial scale	Activated sludge samples were collected from the aerated tanks of the Ribe and Haderslev WWTPs on October 22, 2021, Klosterneuburg WWTP on June 1, 2022. Agricultural soil was collected from 48°11'17.9"N 14°15'16.5"E on May 9, 2023.
Data exclusions	No data was excluded from analysis.
Reproducibility	All performed experiments are reported - even if they show different behaviour in one replicate.
Randomization	Biological triplicates, quadruplicates or quintuplicates per treatment were considered as one experimental group.
Blinding	Blinding was not applicable as the study does not involve living animal or human individuals.

Did the study involve field work? ☒ Yes ☐ No

Field work, collection and transport

Field conditions	Activated sludge from three wastewater treatment plants were sampled. Agricultural soil was sampled from 48°11'17.9"N 14°

Field conditions	15'16.5"E under mild weather conditions.
Location	Ribe, Denmark (GPS: 55.33, 8.74), Haderslev, Denmark (GPS: 55.25, 9.51), Klosterneuburg, Austria (GPS: 48.29, 16.34), Agricultural soil 48°11'17.9"N 14°15'16.5"E
Access & import/export	Sampling of the wastewater treatment plants happened under the supervision of the respective facility management personnel. Living cultures did not cross any borders, they were incubated and sub-sampled in local laboratories. Austrian agricultural soil was sampled and all experiments were performed in Austria, which does not require specific permits.
Disturbance	Sampling the tanks of the wastewater treatment plants or the agricultural soils (longterm field experiment from AGES Austrian Agency for Health and Food Safety) did not cause disturbance to their microbial communities.

Reporting for specific materials, systems and methods

We require information from authors about some types of materials, experimental systems and methods used in many studies. Here, indicate whether each material, system or method listed is relevant to your study. If you are not sure if a list item applies to your research, read the appropriate section before selecting a response.

Materials & experimental systems

n/a	Involved in the study	
☒	☐	Antibodies
☒	☐	Eukaryotic cell lines
☒	☐	Palaeontology and archaeology
☒	☐	Animals and other organisms
☒	☐	Clinical data
☒	☐	Dual use research of concern
☒	☐	Plants

Methods

n/a	Involved in the study	
☒	☐	ChIP-seq
☒	☐	Flow cytometry
☒	☐	MRI-based neuroimaging

Plants

Seed stocks	not applicable
Novel plant genotypes	not applicable
Authentication	not applicable
